# Perceptual organization and visual awareness: the case of amodal completion

**DOI:** 10.3389/fpsyg.2023.1201681

**Published:** 2023-08-17

**Authors:** Ruth Kimchi, Dina Devyatko, Shahar Sabary

**Affiliations:** ^1^Department of Psychology, University of Haifa, Haifa, Israel; ^2^Institute of Information Processing and Decision Making, University of Haifa, Haifa, Israel

**Keywords:** amodal completion, visual awareness, perceptual organization, global completion, local completion, symmetry, good continuation, color-opponent flicker (COF)

## Abstract

We investigated the involvement of visual awareness in amodal completion, and specifically, whether visual awareness plays a differential role in local versus global completion, using a primed shape discrimination paradigm and the color-opponent flicker technique to render the prime invisible. In four experiments, participants discriminated the shape of a target preceded by a partly occluded or a neutral prime. All primes were divergent occlusion patterns in which the local completion is based on good continuation of the contours at the point of occlusion and the global completion is based on maximum symmetry. The target corresponded to the shape that could arise as a result of local or global completion of the occluded prime. For each experiment with an invisible prime we conducted a version with a visible prime. Our results suggest that local completion, but not global completion, of a partly occluded shape can take place in the absence of visual awareness, but apparently only when the visible occluded shape generates a single, local completion. No completion, either local or global, appears to take place in the absence of visual awareness when the visible occluded shape generates multiple completions. The implications of these results to the differential role of visual awareness in local and global completions and to the relationship between multiple completions and unconscious amodal completions are discussed.

## 1. Introduction

Objects in our environment are often partly occluded by other objects or by themselves. Consequently, the input to our visual system is fragmented, yet we perceive our environment as a coherent scene with complete and whole objects. The visual system apparently fills in the incomplete parts of occluded objects, and does it rapidly and effortlessly. This filling in of contours and surfaces behind occluders has been referred to as amodal completion (Michotte et al., 1964,^1991^; see van Lier and Gerbino, 2015, for a review).

Two types of completions have been identified, local, and global. Local completion is based on local contour properties, mainly in line with the Gestalt principle of good continuation (Wertheimer, 1938) – a smooth connection between the visible contours of the occluded object (e.g., Kellman and Shipley, 1991; Wouterlood and Boselie, 1992; Fantoni and Gerbino, 2003). Global completion is based on global shape properties like symmetry and regularity (e. g., Buffart et al., 1981; Sekuler et al., 1994; van Lier et al., 1994, 1995b), fitting with the Gestalt Law of Prägnanz (Koffka, 1935).

In some occlusion patterns, referred to as convergent occlusion patterns, the local and global completions converge toward the same shape, whereas in the divergent occlusion patterns local and global completions yield different shapes (e.g., van Lier et al., 1995b; de Wit et al., 2006; Hazenberg et al., 2014). Research using various paradigms has demonstrated that indeed both local and global completions can be generated and the two completions can be competitive or one can prevail (Sekuler et al., 1994; van Lier et al., 1995a,b; van Lier and Wagemans, 1999; Plomp and van Leeuwen, 2006; Hazenberg et al., 2014).

Under certain conditions amodal completion appears to be cognitively impenetrable, as in the famous Kanizsa’s “horse illusion” (Kanizsa, 1979), in which partly occluded horses tend to be interpreted as a single elongated horse even though it conflicts with our knowledge. Recent studies, however, demonstrated that amodal completion can be influenced by familiarity and knowledge (Hazenberg et al., 2014; Hazenberg and van Lier, 2016; Yun et al., 2018).

Here we aim to examine whether amodal completion can take place in the absence of visual awareness, and specifically, whether visual awareness plays a differential role in local versus global completion.

Research has recently addressed the question whether visual awareness of the stimulus is needed for it to be perceptually organized (e.g., Schwarzkopf and Rees, 2011; Montoro et al., 2014; Kimchi et al., 2018; Sabary et al., 2020). The results suggest that it depends on the perceptual organization processes under study, which is perhaps not surprising in light of the evidence that perceptual organization is a multiplicity of processes that vary in time course, developmental trajectory and attentional demands (e.g., Kimchi, 1998, 2000, 2009; Behrmann and Kimchi, 2003; Kimchi et al., 2005), and on the methods used to suppress the stimulus from awareness as they differ in the level at which the suppression takes place (e.g., Breitmeyer, 2015; Moors et al., 2016; Kimchi et al., 2018). Of particular relevance to the present article, grouping based on mirror symmetry was found to require visual awareness (Devyatko and Kimchi, 2020). Using a priming paradigm and a sandwich masking as an invisibility-inducing method, Devyatko and Kimchi presented participants with masked prime and a clearly visible target, which could be congruent or incongruent with the prime in symmetry. On each trial, the participants performed a two-alternative discrimination task on the target, and then rated the visibility of the prime on a subjective visibility four-point scale. Subjectively invisible primes failed to produce response priming, suggesting that symmetry detection may depend on visual awareness. This finding may suggest that global completion, to the extent that it is based on global symmetry, cannot take place in the absence of visual awareness. We note, however, that the stimuli in Devyatko and Kimchi’s study were quite minimal – composed of just two vertical symmetric or asymmetric lines, thus having just one axis of symmetry when symmetrical. It is possible that in the presence of multiple axes of symmetry unconscious global completion can occur.

Also relevant to the present article are the findings from studies examining the relationship between visual awareness and another type of perceptual completion – modal completion. In modal completion the completed object has sensory qualities, as, for example, the Kanizsa’s illusory triangle (Kanizsa, 1979), in which the observer perceives illusory contours and a bright surface in areas of the stimulus where there is no actual luminance discontinuity. There is little evidence that illusory contours can be formed in the absence of visual awareness. No perception of illusory contours was found when Kanizsa-type inducers were suppressed from awareness by binocular rivalry (Sobel and Blake, 2003), continuous flash suppression (CFS) (Harris et al., 2011), sandwich masking and counter-phase flickering (Banica and Schwarzkopf, 2016). In contrast, Wang et al. (2012), using breaking continuous flash suppression (b-CFS), found that a Kanizsa triangle emerged from suppression significantly faster than a control stimulus, presumably suggesting formation of illusory contours without awareness (but see Moors et al., 2016), and Jimenez et al. (2017) found priming by illusory figure masked by sandwich masking when the prime-mask SOA was 53 ms, but not when it was 23 ms. Thus, the results provide somewhat inconsistent evidence. Furthermore, the question whether amodal and modal completions share the same underlying mechanisms, as suggested by the “identity hypothesis” (Kellman and Shipley, 1991; Shipley and Kellman, 1992), or they have different mechanisms (Anderson et al., 2002; Singh, 2004; Anderson, 2007a), has been a matter of a furious debate (e.g., Kellman et al., 2007; Anderson, 2007b), and the controversy continues (see van Lier and Gerbino, 2015, for a discussion). Therefore we cannot draw clear predictions from the findings concerning the relationship between modal completion and visual awareness to the one between amodal completion and visual awareness.

To the best of our knowledge, Emmanouil and Ro’s (2014) study is the only one to date that attempted to examine whether amodal completion can take place in the absence of visual awareness. Emmanouil and Ro examined the effect of invisible shape primes (a circle in Experiment 1 and a square in Experiment 2) on discrimination of a visible target. Invisibility was induced by metacontrast (Experiment 1) and backward masking (Experiment 2). They found that occluded and unoccluded primes produced a similar pattern of priming, suggesting that the invisible occluded primes were amodally completed. Note that the occluded patterns used by Emmanouil and Ro were highly familiar, convergent occlusion patterns, making it difficult to generalize their results to less familiar and to divergent occlusion patterns. More importantly, there are some concerns associated with Emmanouil and Ro’s study, mainly regarding their prime identification task used for testing the visibility of the primes. The prime identification task in Experiments 1 and 2 included a target and the participants were instructed to ignore it. Not only could the to-be-ignored target bias perception of the prime, but identification of the prime could be susceptible to memory. Furthermore, in Experiment 1, a complete circle was considered as a “correct” response in the occluded and control prime conditions, thus actually testing whether the supposedly invisible prime was completed, rather than testing the visibility of the prime *per se*. In Experiment 2, the invisibility of the prime was questionable because prime identification was significantly above chance. Thus, the absence of a clear evidence for the invisibility of the prime in both experiments casts doubt on the interpretation of the observed results as indicating amodal completion without awareness.

In the present study, we used a priming paradigm in which participants discriminated the shape of a target preceded by a partly occluded or a neutral prime. The partly occluded primes were divergent occlusion patterns adapted from Sekuler et al. (1994) and Plomp and van Leeuwen (2006). The target corresponded to the shape that could arise as a result of a local or a global completion of the partly occluded prime. The prime was suppressed from awareness by a modification of the color-opponent flicker (COF) method developed by Hoshiyama et al. (2006a,b). This method allows to present the prime for as long as required, and the luminance and contrast of the visual stimulus remain constant during the presentation period. This is important because amodal completion takes between 75 to 250 ms to complete, depending on the amount of occlusion and on the experimental task (Sekuler and Palmer, 1992; Murray et al., 2001; Guttman et al., 2003), so that backward masking commonly used to study unconscious processing, in which the prime is briefly presented (~40 ms), cannot be used. Thus, we presented the prime for 300 ms and ensured the invisibility of the prime during the presentation time.

Awareness of the prime was assessed by an objective visibility test, using a prime visibility task. Unconscious completion of the prime was measured as the difference between response to the target after the occluded prime and the neutral prime (*priming effect*). We reasoned that if the occluded prime is completed such that it is the same as the target, then performance after the occluded prime is expected to be better than after the neutral prime.

## 2. General methods

Each experiment included two parts. In the first part, participants performed the priming task; in the second part, following the completion of the first part, the participants performed the visibility task.

For each experiment with invisible prime we conducted a version with a visible prime to ensure that the primes and procedure that we used allow for amodal completion when the primes are visible.

### 2.1. Participants

Participants in all the experiments were students at the University of Haifa and were paid or granted a course credit for participation. All participants provided informed consent to a protocol approved by the Ethics Committee of the University of Haifa. All participants had normal vision and normal color vision and none, except for three, participated in more than one experiment. The sample size for the invisible experiments was calculated on the basis of an *a priori* power analysis (G*Power 3.1; Faul et al., 2007) to detect priming effects, given a moderate effect size (0.50), α = 0.05 and 80% power. The sample size for the visible experiments was based on previously reported sample sizes in studies investigating amodal completion with priming paradigms (Sekuler et al., 1994; van Lier et al., 1995b; Hazenberg et al., 2014).

### 2.2. Apparatus

The experiments took place in a dimly lit room. All stimuli were generated using Matlab R2014a and Psychophysics Toolbox^1^ and were presented on a 20″ sgi color monitor (C22BW711,1024 × 768 resolution, 100 Hz refresh rate) attached to a Mac Pro Late 2013 (3.7 GHz Quad-Core Intel Xeon ES). Responses were collected via Apple keyboard A1243emc 2,171. Participants viewed the stimuli at a distance of 57 cm with their head supported by a chin rest.

### 2.3. Stimuli

**Figure 1:**
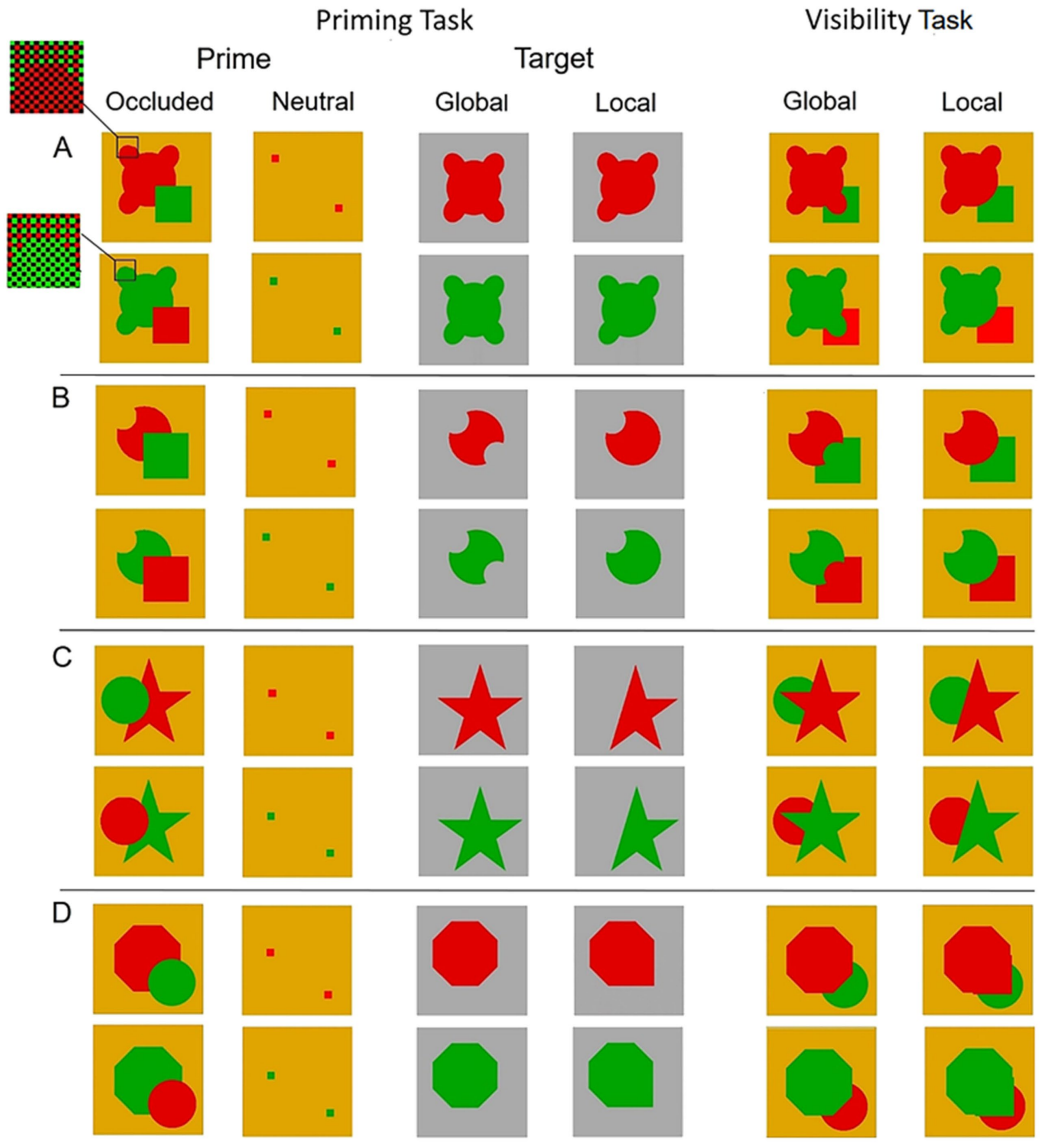
The primes and targets in the priming task, and the primes in the visibility task, used in **(A)** Experiment 1, **(B)** Experiment 2, **(C)** Experiment 3 and **(D)** Experiment 4. The primes were drawn in red or green on a red-and-green checkerboard background and covered with black mesh (see text for details).

The prime stimuli were partly occluded shapes (occluded primes), and neutral primes comprised of two small squares (side: 0.25°) randomly placed within the area occupied by the partly occluded prime stimulus. The prime stimuli were drawn in red (R,G,B: 255,0,0; 13.8 cd/m2; x, y: 0.620, 0.348) and in green (R,G,B: 0,165,0; 13.8 cd/m2; x, y: 0.290, 0.594). When the color of the occluded shape was red, the color of the occluder was green, and when the color of the occluded shape was green the color of the occluder was red. Hereafter, the color of the occluded shape is used for referring to the color of the prime stimulus. The prime stimuli were drawn on a red-and-green checkerboard background (9° X 9°) and covered with a black mesh (Figure 1; see, Hoshiyama et al., 2006a,b). The average amount of contour and surface area occlusion was 20 to 25%.

All primes were divergent occlusion patterns; their local completion is always based on good continuation of the contours at the point of occlusion, and the global completion is based on maximum symmetry.

There were two types of targets corresponding to the two different shapes that could arise as a result of global and local completions of the occluded prime. The targets were presented on a grey (R,G,B: 170,170,170) background.

The stimuli for the visibility task corresponded to the global and local completions of the occluded primes with the occluder placed behind the figure.

The primes and targets in the priming task and the primes in the visibility task for Experiments 1–4 are presented in Figures 1A–D, respectively.

### 2.4. Procedure and design

#### 2.4.1. Invisible prime experiments (Experiments 1a–4a)

These experiments were designed to examine whether amodal completion can take place in the absence of visual awareness. The prime was rendered invisible by means of a modification of the Color-Opponent Flicker (COF) method developed by Hoshiyama et al. (2006a,b). When two isoluminant opponent colors, for example, red and green, alternate at frequencies above the flicker fusion threshold (∼30 Hz), the two colors fuse such that one uniformly yellow color is perceived (e.g., Schiller and Logothetis, 1990). In the conventional COF method, the red and green colors must be isoluminant. Hoshiyama et al.’s (2006a,b) modification of covering the figures with a black mesh prevents one color from being directly adjacent to another color such that no edges, caused by the difference in luminosity between two colors during COF, are produced, and consequently there is no need to strictly control the two colors for isoluminosity.

In each experiment, participants first undertook the masked priming task and following its completion they performed the visibility task.

##### 2.4.1.1. Priming task

**Figure 2:**
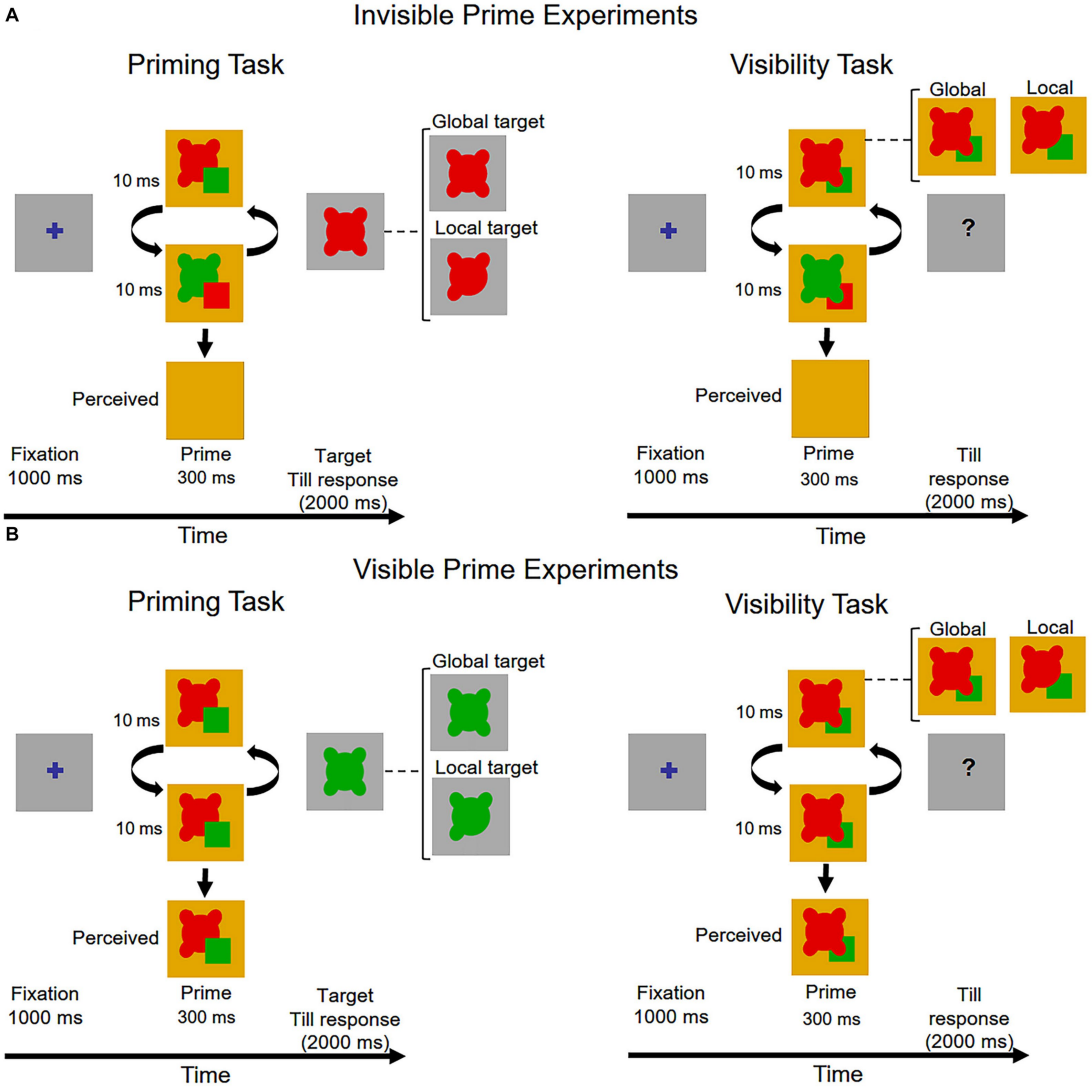
The sequence of events in a trial in the priming task (left panel) and in the visibility task (right panel) in **(A)** the invisible prime experiments and **(B)** the visible prime experiments. The figure depicts the prime and the global target of Experiment 1 (see text for details).

The sequence of events in a trial in the priming task is shown in Figure 2A (left panel). Each trial started with the presentation of a fixation mark (0.5° × 0.5° blue cross, R,G,B, 0,0,255) at the center of the screen for 1,000 ms. Then a pair of primes was presented alternately (e.g., red-green-red green…) at 100 Hz (10 ms presentation of each image) for 300 ms. Previous research have suggested that given the amount of occlusion that we used, presentation time of 300 ms is clearly sufficient for perceptual completion to take place (Murray et al., 2001; Guttman et al., 2003). The red and green primes fused and a uniform dark yellowish color was perceived. In half of the trials the alternated primes started with the red prime and in the other half with the green prime. Following the prime, a visible target (local or global) appeared and remained on the screen until the participant responded or 2,000 ms had elapsed. The color of the target shape was opposite to the color of the last prime during the COF sequence. Participants had to indicate the shape of the target by pressing one of two keys (“local shape” key or “global shape” key) with their dominant hand as fast as possible while avoiding making mistakes (Participants were told that the color of the shapes was irrelevant.)

All the combinations of prime type (occluded, neutral), first prime color in the alternated sequence (red, green) and target (global, local) were presented with equal frequency in a random order. Each participant completed 240 trials with five self-administrated breaks, preceded by 16 practice trials. During the practice, an auditory tone provided immediate feedback after an incorrect response or when 2,000 ms had elapsed with no response.

##### 2.4.1.2. Visibility task

After completing the masked priming task participants performed the visibility task. The sequence of events in a trial, presented in Figure 2A (right panel), was similar to that of the priming task except that no target was presented after the presentation of the masked prime; instead of the target a question mark appeared and stayed on the screen till response. The prime stimuli were a pair of red and green global primes or a pair of red and green local primes (see Figure 1). Participant had to indicate the shape of the prime by pressing one of two keys (“global shape” key or “local shape” key), and were instructed to guess the prime shape if the prime was invisible. All the combinations of the two prime types (global, local) and the first prime color (red, green) were presented with equal frequency in a random order. There were 40 trials preceded with 4 practice trials. No feedback was provided in the practice trials.

After completing the whole experiment, the participants were tested with two cards (# 70 and # 74) from Ishihara’s Test for Color Deficiency in order to make sure they had normal color vision.

#### 2.4.2. Visible prime experiments (Experiments 1b–4b)

For each of the invisible prime experiment we conducted a version in which the prime was visible. The visible prime experiment was similar to the corresponding invisible prime experiment, except that the colors of the primes did not alternate during a trial; either a red prime or a green prime was presented at 100 Hz (10 ms presentation of each image) for 300 ms, resulting in a visible prime. The sequence of events in a trial in the priming task and in the visibility task is presented in Figure 2B.

Apparatus, procedure and design were the same for all experiments.

### 2.5. Data analysis

All reaction time (RT) summaries and analyses are based on participants’ mean RTs for correct responses. Trials with RTs shorter than 200 ms or longer than 1,600 ms were excluded from the analyses (less than 1% in all experiments). Because instructions to the participants emphasized both speed and accuracy, and to simplify presentation and analyses, we used an inverse efficiency (IE) score (mean correct RT divided by proportion of correct responses) as the dependent measure (Townsend and Ashby, 1978, 1983). Using IE in the present data analyses was appropriate given the high accuracy rate, which exceeded 94% in all our experiments, and the similar pattern of results for RT and accuracy measures (Bruyer and Brysbaert, 2011; Vandierendonck, 2017, 2018). Repeated measures ANOVAs were used to analyze the IE data. All ANOVAs were calculated using SAS (version 9.4). See the Supplementary materials for analyses of accuracy and RT separately.

When null effects were theoretically important, i.e., inferring that awareness may be necessary for amodal completion to occur, we also evaluated evidence in favor of the null hypothesis by computing the Bayes factor (BF10) in a Bayesian paired t-test, using JASP statistical software (www.jasp-stats.org) and a Cauchy prior centered on zero (scale = 0.707).

## 3. Experiment 1

### 3.1. Participants

Twenty-seven individuals (24 females and 3 males, 4 left-handed, age ranged 18–29, *M* = 23.2) participated in Experiment 1a (Invisible-prime experiment), and 18 individuals (13 females and 5 males, 3 left-handed, age ranged 19–31, *M* = 23.1) participated in Experiment 1b (Visible-prime experiment).

### 3.2. Stimuli

The basic shape was a symmetrical shape consisting of a large circle (3.95° in diameter) and four elliptical protrusions and containing two axes of symmetry. The overall size of the shape was 4.8° X 4.2°. The symmetrical shape was partly occluded by a 2.7° × 2.7° square, constituting the occluded prime (Figure 1A). The global completion of the occluded shape resulted in the symmetrical shape, whereas the local completion by good continuation of the visible contours of the occluded shape resulted in a different shape with three elliptical protrusions and one axis of symmetry. The two targets corresponded to the two shapes that resulted from global and local completions, respectively (Figure 1A). The centers of both targets were moved 0.53° below and to the right from the center of the occluded prime in order to avoid full overlapping of prime’s and targets’ contours. Two primes were used in the visibility task, designated as global and local, which were produced by placing the occluding square behind the shapes generated by the global and local completions (Figure 1A).

### 3.3. Results and discussion

#### 3.3.1. Experiment 1a: invisible prime

**Figure 3:**
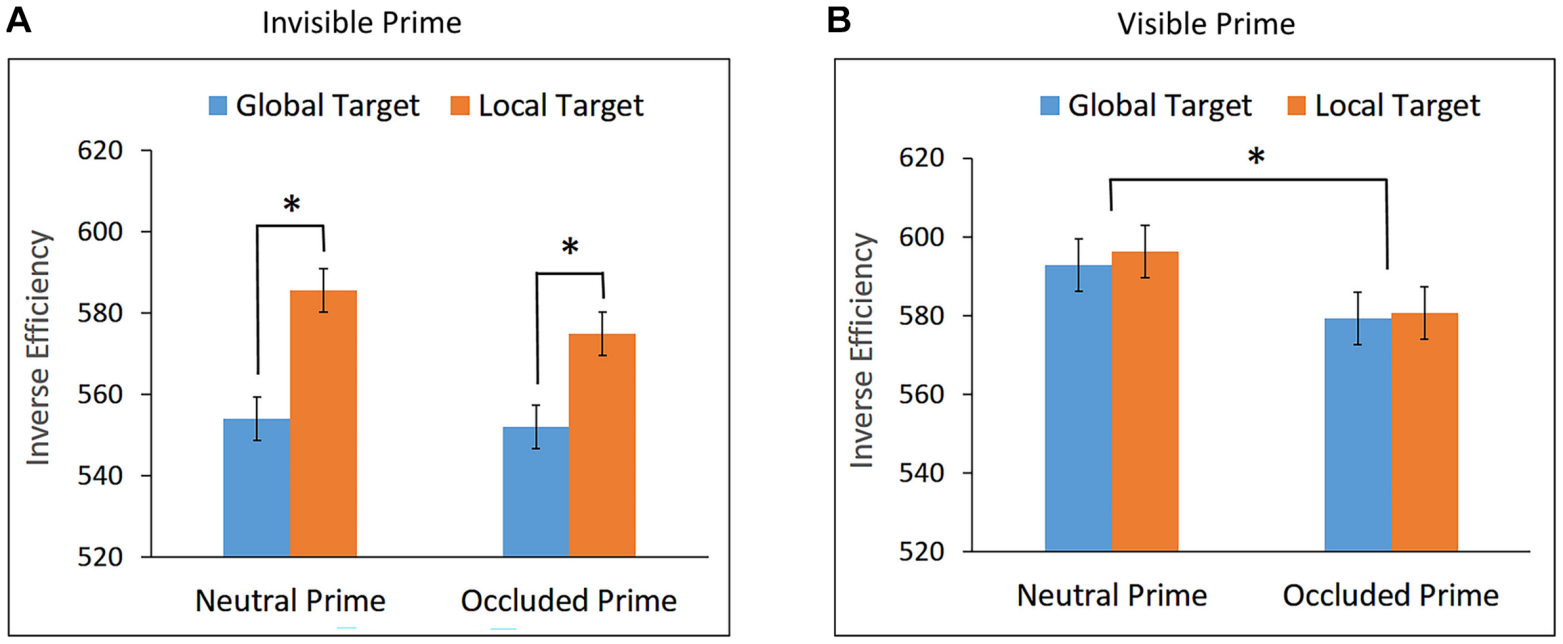
Inverse Efficiency (IE) scores for global and local targets in the neutral and occluded prime conditions in **(A)** Experiment 1a – Invisible prime, and **(B)** Experiment 1b – Visible prime. Error bars represent within subjects ± SEM.

Trials in which RT was longer than 1,600 ms or shorter than 200 ms were excluded from the analyses (0.34%). Mean RTs and mean accuracy (AC) for global and local targets in the neutral and occluded prime conditions are presented in Table 1. Mean IE scores are presented in Figure 3A.

**Table 1:** Mean RTs (ms) and mean AC (%) for global and local target as a function of prime condition (neutral and occluded) for each experiment.

	Global target				Local target			
	Neutral prime		Occluded prime		Neutral prime		Occluded prime	
	RT	AC	RT	AC	RT	AC	RT	AC
Experiment 1a	540.89	97.46	539.18	97.59	555.88	94.75	553.41	96.17
Experiment 1b	581.82	97.96	561.67	96.94	584.18	97.15	567.17	97.31
Experiment 2a	536.62	95.61	548.49	97.03	550.26	95.92	542.28	96.54
Experiment 2b	545.63	96.85	546.54	95.37	542.75	96.48	521.46	95.83
Experiment 3a	471.56	97.71	472.26	97.28	466.74	97.59	472.09	97.35
Experiment 3b	492.15	96.57	483.71	96.94	489.76	96.85	472.49	97.50
Experiment 4a	471.27	98.02	474.39	98.63	468.91	97.84	469.29	97.22
Experiment 4b	504.96	97.68	492.64	98.24	498.63	97.02	502.37	96.66

Performance in the visibility task did not differ significantly from chance, mean accuracy = 51.85%, *t*(26) = 1.43, *p* = 0.1665, indicating that COF rendered the prime invisible.

The repeated measures ANOVA with prime (neutral and occluded) and target (global and local) as within-subject factors, conducted on the IE scores, revealed a main effect of target, *F*(1,26) = 10.11, *p* = 0.0038, η_p_^2^ = 0.28. As can be seen in Figure 3A, performance for the global target was better than performance for the local target, both in the neutral prime condition, *t*(26) = 2.972, *p* = 0.003, Cohen’s d = 0.572, and in the occluded prime condition, *t*(26) = 2.421, *p* = 0.011, Cohen’s d = 0.455.

The main effect of prime, *F*(1,26) = 1.78, *p* = 0.1941, and the interaction between target and prime conditions, *F* < 1, were not significant, showing no indication of priming effects. Bayesian paired t-tests showed that the evidence provides substantial support for the null hypothesis for global priming, BF10 = 0.275, and is inconclusive for local priming, BF10 = 0.742.

These results are seen to suggest that no completion, either local or global, took place when the occluded prime was invisible.

#### 3.3.2. Experiment 1b: visible prime

Trials in which RT was shorter than 200 ms or longer than 1,600 ms were excluded from the analyses (0.65%). Mean RTs and mean AC for global and local targets in the neutral and occluded prime conditions are presented in Table 1. Mean IE scores are presented in Figure 3B.

Performance in the visibility task was significantly above chance, mean accuracy = 96.94%, *t*(26) = 71.49, *p* < 0.0001, confirming that the prime was visible.

The repeated measures ANOVA conducted on the IE data showed no main effect of target, *F* < 1. The effect of prime was significant, *F*(1,17) = 4.84, *p* = 0.0419, η_p_^2^ = 0.22, indicating that performance was better when the targets appeared after the occluded prime than after the neutral prime. As can be seen in Figure 3B, this facilitation effect did not interact with target, *F* < 1, suggesting global and local priming effects, which did not differ significantly in magnitude.

These results suggest that when the partly occluded prime was visible, both global and local completions were generated and no completion was significantly preferred over the other. Multiple completion were previously observed with different stimuli (van Lier et al., 1995a,b), and van Lier et al. suggested that the preference for a global or a local completion is the consequence of a competition between interpretations.

Interestingly, Sekuler et al. (1994) used a stimulus similar to ours and a primed matching paradigm, and found a clear global completion. What may account for the discrepancy in the results? First, the stimuli used by Sekuler and colleagues were “square like,” having 4 axes of symmetry, whereas the stimuli we used were elongated and had only 2 axes of symmetry. Second, although the stimuli were very similar, they could differ in the amount of occlusion. In the absence of specific details we can just eyeball and it seems that the amount of contour occlusion in Sekuler et al. was somewhat larger, such that the connection between the visible contours of the occluded object was smoother in our stimulus, increasing the likelihood of local completion; thus a local completion was generated and competed with the global completion. Third, the presentation of the prime was different, and so was the task (target shape discrimination vs. same-different judgments). These differences could in principle affect the pattern of results, but further research is required in order to understand how.

In contrast to the multiple completions observed for the visible prime, the results for the invisible prime (Experiment 1a) showed no completion, either local or global, suggesting that amodal completion cannot take place in the absence of visual awareness.

## 4. Experiment 2

### 4.1. Participants

Twenty-seven individuals (17 females and 10 males, 3 left-handed, age ranged 18–34, *M* = 24.1) participated in Experiment 2a, and 18 individuals (11 females and 7 males, 3 left-handed, age ranged 19–30, *M* = 22.9) participated in Experiment 2b.

### 4.2. Stimuli

The basic shape was a symmetrical shape generated by a large circle (4.5° in diameter) with a circular cut-off in the upper left side and in the upper right side (2.25° in diameter each), the center of which located at the large circle circumference. The shape contained two axes of symmetry. The symmetrical shape was partly occluded by a 3.75° × 3.75° square, constituting the occluded prime (Figure 1B). The global completion of the occluded shape resulted in the symmetrical shape. The local completion by good continuation of the visible contours of the occluded shape resulted in a different shape of a circle with a single cut-off in the upper left side, and contained one axis of symmetry. The two targets corresponded to the two shapes that resulted from global and local completions (Figure 1B). The centers of the large circles in both targets were 0.8° below the center of the prime’s large circle in order to avoid full overlapping of prime’s and targets’ contours. Two primes were used in the visibility task, global and local, which were produced by placing the occluding square behind the global and local completed shapes (Figure 1B).

### 4.3. Results and discussion

#### 4.3.1. Experiment 2a: invisible prime

**Figure 4:**
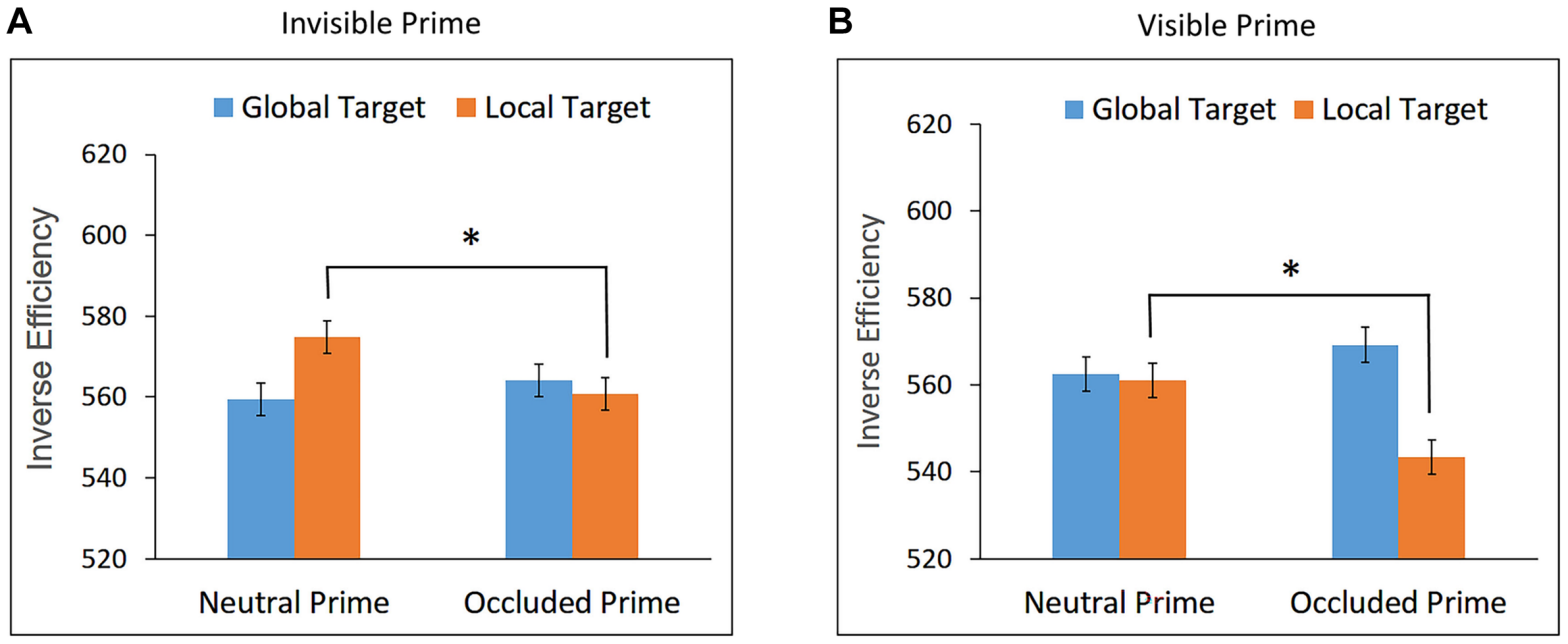
Inverse Efficiency scores for global and local targets as a function of prime condition (occluded and neutral) in **(A)** Experiment 2a – Invisible prime, and **(B)** Experiment 2b – Visible prime. Error bars represent within subjects ± SEM.

Trials with RTs shorter than 200 ms or longer than 1,600 ms were excluded from the analysis (0.45%). Mean RTs and mean accuracy for global and local targets in the neutral and occluded prime conditions are presented in Table 1. Mean IE scores are presented in Figure 4A.

Performance in the visibility task was at chance, mean accuracy = 51.76%, *t* = 0.90, *p* = 0.3777, confirming that the prime was invisible.

The repeated measures ANOVA conducted on the IE data showed no main effects of target, *F* < 1, and prime, *F* < 1. The interaction between target and prime, however, was significant, *F*(1,26) = 5.15, *p* = 0.0318, η_p_^2^ = 0.17. As can be seen in Figure 4A, performance for the local target was better following the occluded prime than the neutral prime, *t*(26) = 1.949, *p* = 0.031, Cohen’s *d* = 0.375, indicating local priming. No such effect whatsoever was observed for the global target, *t*(26) = −0.889, *p* = 0.809; a Bayesian paired t-test showed that the evidence provides substantial support for the null hypothesis, BF10 = 0.117.

To rule out the possibility that the observed local priming was due to the performance of participants for whom the prime was visible, we calculated, for each participant, the local priming score and the visibility score (i.e., accuracy in the visibility task) and examined whether there is a positive correlation between these two scores. The analysis yielded no significant correlation, *r* = −0.284, *p* = 0.924; Bayesian correlation showed that the evidence provided substantial support for the null hypothesis, BF10 = 0.106.

These results suggest that local completion of a partly occluded object can take place in the absence of visual awareness.

#### 4.3.2. Experiment 2b: visible prime

Trials with RTs shorter than 200 ms or longer than 1,600 ms were excluded from the analysis (0.83%). Mean RTs and mean accuracy for global and local targets in the neutral and occluded prime conditions are presented in Table 1. Mean IE scores are presented in Figure 4B.

Performance in the visibility task was significantly above chance, mean accuracy = 94.03%, *t*(17) = 30.76, *p* < 0.0001, confirming that the prime was visible.

The repeated measures ANOVA conducted on the IE data showed no significant main effects of prime, F < 1, nor of target, *F*(1,17) = 1.57, *p* = 0.2274. The interaction between target and prime, however, was significant, *F*(1,17) = 7.78, *p* = 0.0127, η_p_^2^ = 0.31. As can be seen in Figures 4B, a priming effect for the local target was observed, *t*(17) = 3.028, *p* = 0.004, Cohen’s *d* = 0.717, suggesting local completion. No priming effect was observed for the global target, *t*(17) = −0.715, *p* = 0.758; Bayesian paired t-test showed that the evidence provides substantial support for the null hypothesis, BF10 = 0.155.

In contrast to our results, Plomp and van Leeuwen (2006) found some preference for global completion for a similar stimulus. However, the procedures of their study and the current study are quite different, making the comparison between the two studies difficult.

Inspection of our Figure 4 shows that the pattern of results with the invisible prime (Experiment 2a) was similar to the one with the visible prime (Experiment 2b): a facilitation for the response to the local target following occluded prime, and no such facilitation whatsoever for the response to the global target. These results suggest that local completion was taking place both in the presence and in the absence of visual awareness.

Comparing the results of Experiments 1 and 2 reveals an interesting pattern. In Experiment 1, no completion was observed for the invisible prime (Experiment 1a), which, when visible, generated multiple completions (Experiment 1b). In Experiment 2, local completion was observed for the invisible prime (Experiment 2a), which, when visible generated a single local completion (Experiment 2b). Presumably, the potential generation of multiple completions versus a single completion may influence whether or not unconscious completion occurs. We return to this issue later.

## 5. Experiment 3

Previous research showed, as noted earlier, that familiarity and knowledge can have an effect on amodal completion (Hazenberg et al., 2014; Hazenberg and van Lier, 2016; Yun et al., 2018). Experiment 3 was designed to examine whether familiarity can influence amodal completion in the absence of awareness. To this end, the prime we used was a partially occluded five-point star (Figure 1C). Familiarity in this case favors the global completion.

### 5.1. Participants

Twenty-seven individuals (18 females and 9 males, 1 left-handed, age ranged 19–34, M = 25.85) participated in Experiment 3a, and 18 individuals (13 females and 5 males, 2 left-handed, age ranged 19–35, *M* = 23.89) participated in Experiment 3b.

### 5.2. Stimuli

The basic shape was a five-point star (6.2° by 6.2° in height and width), containing five axes of symmetry. The star was partly occluded by a circle (3.5° in diameter), constituting the occluded prime (Figure 1C). The global completion of the occluded shape resulted in the star. The local completion by good continuation of the collinear visible contours of the occluded star resulted in a different shape, and contained one axis of symmetry. The two targets corresponded to the two shapes that resulted from the global and local completions (Figure 1C). The centers of both targets were moved 0.5° below and to the right from the center of the occluded prime in order to avoid full overlapping of prime’s and targets’ contours. Two primes were used in the visibility task, global and local, which were produced by placing the occluding circle behind the global and local completed shapes (Figure 1C).

### 5.3. Results and discussion

#### 5.3.1. Experiment 3a: invisible prime

**Figure 5:**
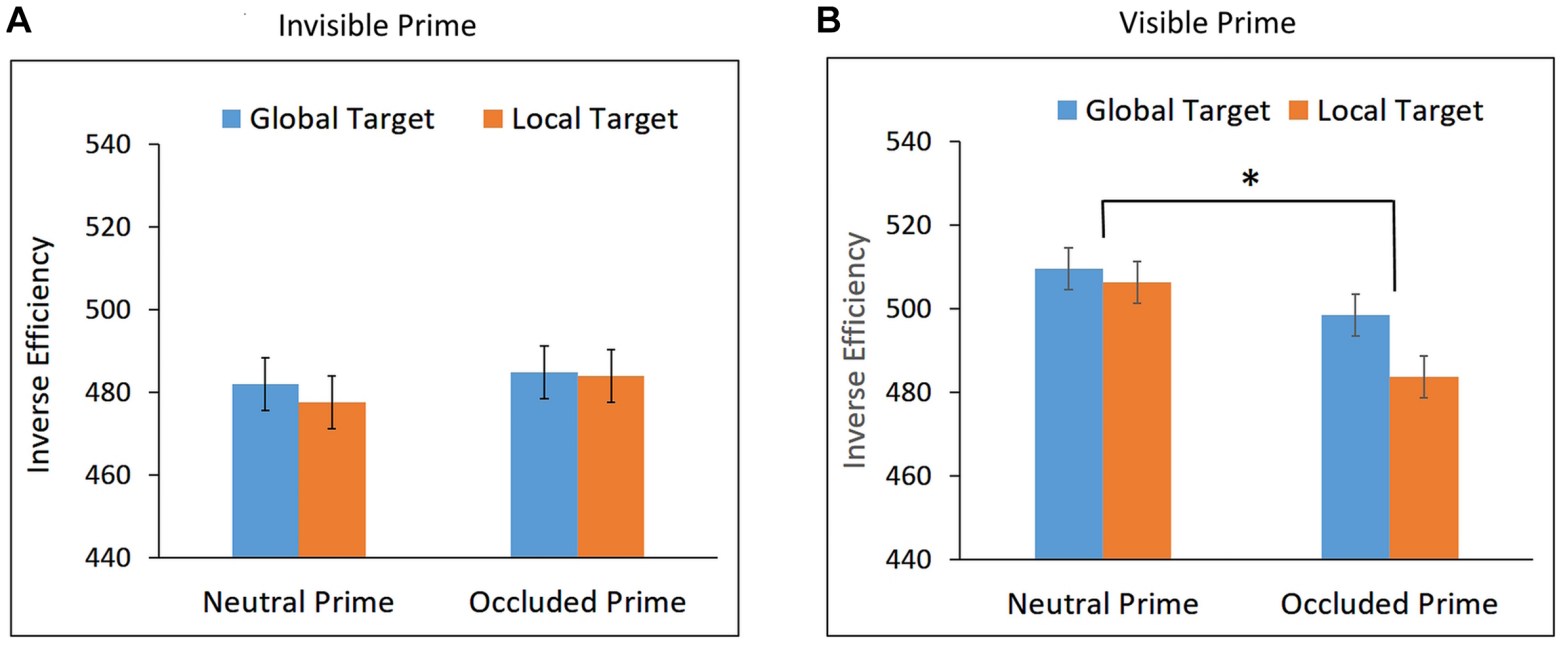
Inverse Efficiency scores for global and local targets as a function of prime condition (occluded and neutral) in **(A)** Experiment 3a – Invisible prime, and **(B)** Experiment 3b – Visible prime. Error bars represent within subjects ± SEM.

Trials with RTs shorter than 200 ms or longer than 1,600 ms were excluded from the analysis (0.22%). Mean RT and mean accuracy for global and local targets in the neutral and occluded prime conditions are presented in Table 1. Mean IE scores are presented in Figure 5A.

Performance in the visibility task did not differ from chance, mean accuracy = 50.37%, *t*(26) = 0.34, *p* = 0.7343, confirming that the prime was invisible.

None of the effects tested by the repeated measures ANOVA, conducted on the IE scores, reached statistical significance: *F* < 1, *F*(1,26) = 3.53, *p* = 0.0714, and *F < 1*, for the effects of target, prime and prime X target interaction, respectively. These results show no indication of global or local priming; Bayesian paired t-tests showed that the evidence provides substantial support for the null hypothesis for the former, BF10 = 0.133, and strong support for the latter, BF10 = 0.080.

These findings indicate that neither local nor global completion took place when the occluded prime was invisible, and suggest that familiarity had no influence on amodal completion in the absence of visual awareness.

#### 5.3.2. Experiment 3b: visible prime

Trials with RTs shorter than 200 ms or longer than 1,600 ms were excluded from the analysis (0.53%). Mean RTs and mean accuracy for global and local targets in the neutral and occluded prime conditions are presented in Table 1. Mean IE scores are presented in Figure 5B.

Performance in the visibility task was significantly above chance, mean accuracy = 96.53%, *t*(17) = 50.07, *p* < 0.0001, confirming that the prime was visible.

The repeated measures ANOVA showed no significant effect of target, *F* < 1. The effect of prime was significant, *F*(1,17) = 11.58, *p* = 0.0034, η_p_^2^ = 0.41, indicating better performance for the targets following an occluded prime than the neutral prime. As can be seen in Figure 5B, this facilitation effect did not interact with target, *F* < 1, suggesting both global and local priming effects, which did not differ significantly in magnitude.

Interestingly, although both familiarity and maximum symmetry favor global completion of the partly occluded star, no preference for global completion was observed in our experiment. The local completion constituted a competing alternative, presumably due to the collinearity of the lines at the point of occlusion. We note that Plomp and van Leeuwen (2006) found some preference for global completion for the star stimulus. However, as noted earlier, the procedures of their study and the current study are quite different, making the comparison between the two studies difficult.

The results of Experiment 3 are similar to the ones of Experiment 1, indicating no completion, either local or global, in the absence of visual awareness for an occluded shape, which when visible generated multiple completions. On the other hand, local completion in the absence of visual awareness was observed for an occluded shape that when visible generated a single local completion (Experiment 2).

No indication of unconscious global completion was found. However, the results of Experiment 2 suggest that in order to reach a clear conclusion regarding the necessity of visual awareness for global completion to occur, unconscious global completion has to be examined with an occluded shape that generates a single global completion when visible. The next experiment was designed to do so.

## 6. Experiment 4

This experiment was designed to examine whether global completion can take place in the absence of visual awareness. To this end we used an octagon partly occluded by a circle. We suspected that the occluded octagon could be a good candidate for generating a single global completion because the amount of symmetry axes – eight axes in the octagon versus only one in the locally completed shape – strengthen the tendency for global completion, whereas the relatively weak connection between the lines at the point of occlusion (the lines meet at 90° angle) weakens the tendency for local completion. We ran first the visible prime version of this experiment, the results of which confirmed our supposition, and then we ran the invisible version. To be consistent with all other experiments reported in this article, we keep the same order, reporting first the invisible prime experiment and then the visible prime one.

### 6.1. Participants

Twenty-seven individuals (19 females and 8 males, 3 left-handed, age ranged 19–36, M = 25.67) participated in Experiment 4a, and 18 individuals (15 females and 3 males, 1 left-handed, age ranged 18–41, *M* = 24.72) participated in Experiment 4b.

### 6.2. Stimuli

The basic shape was an octagon (4.7° × 4.7° in height and width), containing eight axes of symmetry. The octagon was partly occluded by a circle (3.5° in diameter), constituting the occluded prime (Figure 1D). The global completion of the occluded shape resulted in the octagon. The local completion by continuation of the visible contours of the occluded octagon resulted in a different shape, and contained one axis of symmetry. The two targets corresponded to the two shapes that resulted from the global and local completions (Figure 1D). The centers of both targets were moved 0.5° above and to the left from the center of the occluded prime in order to avoid full overlapping of prime’s and targets’ contours. Two primes were used in the visibility task, global and local, which were produced by placing the occluding circle behind the global and local completed shapes (Figure 1D).

### 6.3. Results and discussion

#### 6.3.1. Experiment 4a: invisible prime

**Figure 6:**
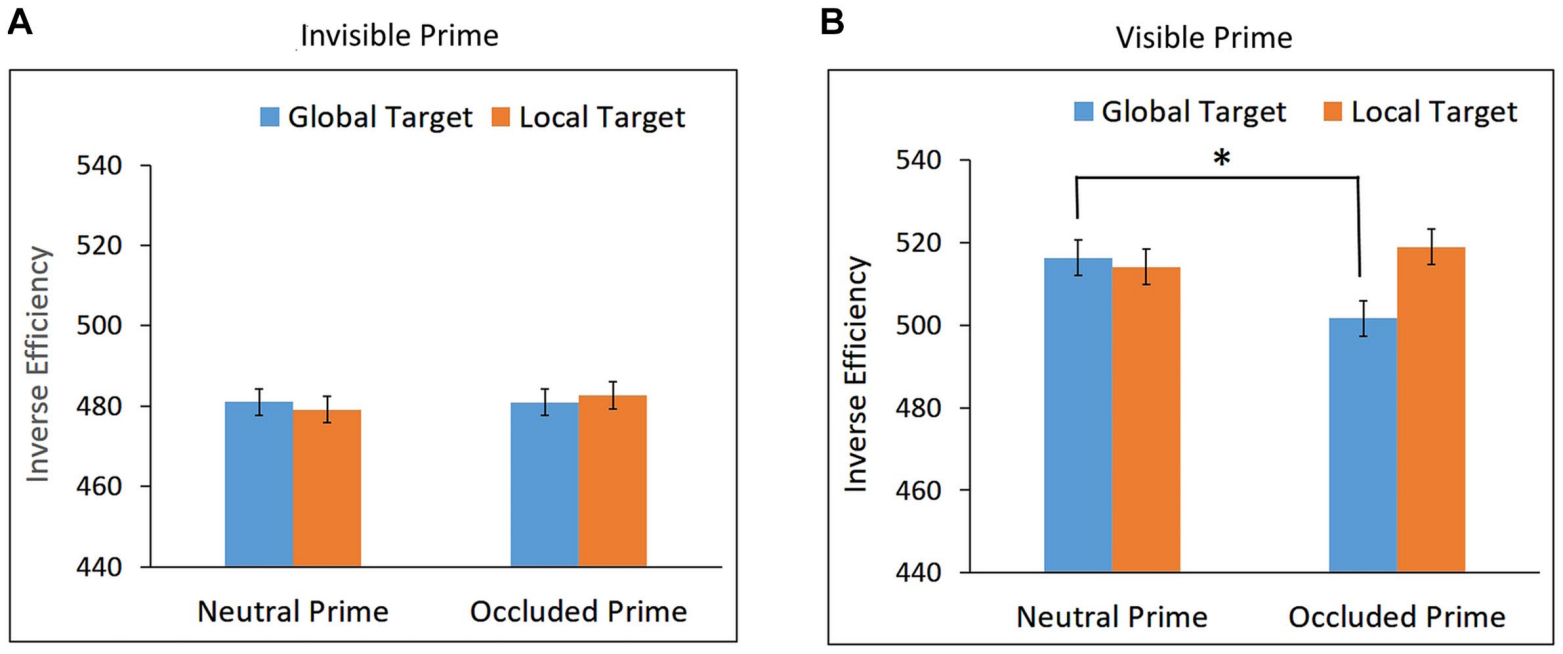
Inverse Efficiency scores for global and local targets as a function of prime condition (occluded and neutral) in **(A)** Experiment 4a – Invisible prime, and **(B)** Experiment 4b – Visible prime. Error bars represent within subjects ± SEM.

Trials with RTs shorter than 200 ms or longer than 1,600 ms were excluded from the analysis (0.15%). Mean RT and mean accuracy for global and local targets in the neutral and occluded prime conditions are presented in Table 1. Mean IE scores are presented in Figure 6A.

Performance in the visibility task did not differ from chance, mean accuracy = 49.72%, *t*(26) = −0.21, *p* = 0.832, confirming that the prime was invisible.

The repeated measures ANOVA showed no significant effect of target, prime, and prime X target interaction, *F*s < 1, indicating no local or global priming. Bayesian paired t-tests showed that the evidence provides substantial support for the null hypothesis, BF10 = 0.116, BF10 = 0.207, for local and global priming, respectively. These findings suggest that neither local nor global completion took place when the occluded prime was invisible.

#### 6.3.2. Experiment 4b: visible prime

Trials with RTs shorter than 200 ms or longer than 1,600 ms were excluded from the analysis (0.39%). Mean RTs and mean accuracy for global and local targets in the neutral and occluded prime conditions are presented in Table 1. Mean IE scores are presented in Figure 6B.

Performance in the visibility task was significantly above chance, mean accuracy = 97.36%, *t*(17) = 61.6, *p* < 0.0001, confirming that the prime was visible.

The repeated measures ANOVA showed no significant effect of target, *F* < 1, nor of prime, *F* < 1. The interaction between target and prime, however, was significant, *F*(1,17) = 5.04, *p* = 0.0383, η_p_^2^ = 0.23. As can be seen in Figures 6B, a priming effect for the global target was observed, *t*(17) = 2.099, *p* = 0.026, Cohen’s d = 0.495, suggesting global completion. No priming effect was observed for the local target, *t*(17) = −0.594, *p* = 0.720; Bayesian paired t-test showed that the evidence provides substantial support for the null hypothesis, BF10 = 0.165.

These results indicate that when the partly occluded octagon was visible, a single global completion was generated. In contrast, no perceptual completion was observed when the partly occluded octagon was invisible.

Thus, the results of Experiment 4 suggest that no global completion can take place in the absence of visual awareness. Assuming that the global completion is based on the overall symmetry of the occluded shape, this finding is in agreement with the finding reported by Devyatko and Kimchi’s (2020) that symmetry-based grouping requires visual awareness, although the stimuli in their study had one vertical axes of symmetry whereas the present octagon had eight axes of symmetry.

In addition, the results of Experiment 4 suggest that generating a single completion is not sufficient for unconscious completion to occur.

## 7. General discussion

In this study we investigated whether amodal completion can take place in the absence of visual awareness, and specifically, whether visual awareness plays a differential role in local versus global completion. To this end we used a primed shape discrimination paradigm in which the prime was rendered invisible by color-opponent flicker (COF; Hoshiyama et al., 2006a,b). All primes were divergent occlusion patterns in which the local completion is always based on good continuation of the contours at the point of occlusion, and the global completion is based on maximum symmetry. The targets corresponded to the two different shapes that could arise as a result of global and local completions of the occluded prime. For each of the invisible prime experiment we conducted a version in which the prime was visible.

The results provide a somewhat complicated picture. No significant local or global priming was observed for invisible occluded primes that generated multiple completions when visible (Experiments 1 and 3), suggesting that no completion, either global or local, can take place in the absence of visual awareness for occluded shapes that, when visible, generate both local and global completions. A significant local priming for an invisible occluded prime was found, suggesting that local completion can occur in the absence of visual awareness, but only for an occluded prime that when visible generates a single local completion (Experiment 2). In contrast, the results showed no global priming for an invisible occluded prime, suggesting that no global completion can take place in the absence of visual awareness, even when the occluded prime generates a single global completion when visible (Experiment 4). In addition, familiarity did not have an effect on unconscious amodal completion (Experiment 3). We note however, that in our study familiarity favored global completion. It would be interesting to examine the influence of familiarity when it favors local completion.

Taken together, the results of Experiments 1–4 are seen to have two important implications. One concerns the role of visual awareness in local versus global completion. The other concerns the relationship between potential multiple completions and unconscious amodal completion. The two are not completely unrelated.

The present results demonstrate that visual awareness plays a differential role in local versus global completion: local completion can take place in the absence of visual awareness, whereas visual awareness is required for global completion to occur. This is perhaps not surprising. Local completion is based mainly on the basic grouping principle of good continuation (Wertheimer, 1938), which plays an important role in contour integration (e.g., Field et al., 1993; Kimchi, 2000; Geisler et al., 2001), processed in the early brain regions V1 and V2 (e.g., Roelfsema, 2006), and appears to operate in the absence of visual awareness (Breitmeyer et al., 2005; Devyatko et al., 2019). Global completion, is based on maximum symmetry. In contrast to good continuation, symmetry produces strong responses only in higher-order regions, especially V4 and LOC (e.g., Sasaki et al., 2005), symmetry-based grouping was found to require visual awareness (Devyatko and Kimchi, 2020; but see, Makin et al., 2023), and the role of symmetry in perceptual organization is not entirely clear because of its interaction with other grouping factors (van der Helm, 2015). For example, a number of researchers argue that grouping by other factors precedes and facilitates grouping by symmetry (see for discussion Machilsen et al., 2009). Also, it was found that organization by collinearity alone suffices for automatic capture of attention by a perceptual object, whereas organization by symmetry alone does not, suggesting that symmetry may play a weaker role than collinearity in the formation of objecthood (Kimchi et al., 2016). Furthermore, according to the Gestaltists, symmetry (like closure) is not a grouping factor *per se*, but rather it plays a critical role in how the perceptual system arrives at a stable, organized structure, with symmetry being particularly crucial in determining figural goodness (Koffka, 1935; Wertheimer, 1938; Palmer, 1991). It is possible that this can be achieved only when the stimulus is consciously perceived.

One may argue that the differential role of visual awareness in local versus global completion supports the view that local and global completions are qualitatively different processes (e. g., Kellman et al., 2005). This view suggests that local completion is a bottom-up process based on stimulus structure whereas global completion is a top-down process, referred to by Carrigan et al. (2016) and Kellman et al. (2005) as “recognition from partial information” (but see Peta et al., 2019 for a critical discussion).

In our opinion, however, the present results do not necessarily suggest that there is a qualitative difference between the processes involved in local and global completions, as both processes can be based on stimulus properties, which can be simple local properties or more complex global properties. This of course does not rule out the possibility of top-down influences such as familiarity and knowledge on amodal completion (e.g., Hazenberg et al., 2014; Yun et al., 2018). But familiarity and knowledge are not to be confused with symmetry and regularity, because the formers depend on the perceiver’s past experience whereas the latter on stimulus structure (see also Peta et al., 2019). Our results are seen to suggest that completion based on simple, local properties can take place in the absence of visual awareness, at least under certain conditions, but completion based on more complex global properties such as symmetry cannot.

The second implication of the present results concerns the relationship between potential multiple completions and unconscious amodal completion. It appears that when there is an unresolved competition between local and global completions, no completion can take place in the absence of visual awareness. Our results also show that a single completion is not sufficient for unconscious completion to occur. Obviously, further research is required in order to get a clearer picture of this relationship. The results of our visible prime experiments show, on the one hand, either a local or a global completion (Experiments 2 and 4), and on the other hand, two competing completions without a local or a global preference (Experiments 1 and 3). It would be interesting to find out what happens in the absence of visual awareness when there is competition between the two completions, but the competition is resolved and one completion prevails. Namely, the question is whether it is the mere presence of a competition or the presence of unresolved competition that requires visual awareness for amodal completion to take place.

Before concluding, we note that unconscious global and local completions need to be explored with different suppression methods, because previous research showed that the extent of information processing without consciousness is also dependent on the invisibility-inducing method – i.e., on the level at which the suppression induced by the method takes place (e.g., Breitmeyer, 2015; Moors et al., 2016; Kimchi et al., 2018).

To summarize, our results suggest that local completion, but not global completion, of a partly occluded shape can take place in the absence of visual awareness, but apparently only when the visible occluded shape generates a single, local completion. No completion appears to take place in the absence of visual awareness when the visible occluded shape generates multiple completions. Further research is required to clarify the relationship between multiple completions and unconscious amodal completion, as well as the effect of familiarity on unconscious completion.

## Data availability statement

The raw data supporting the conclusions of this article will be made available by the authors, without undue reservation.

## Ethics statement

The studies involving humans were approved by the Ethics Committee of the University of Haifa. The participants provided their written informed consent to participate in this study.

## Author contributions

RK: conceptualization, methodology, formal analysis, writing, supervision, funding acquisition. DD and SS: methodology, testing and data collection, formal analysis. All authors approved the submitted version.

## Funding

This research was supported by a grant (grant number 1473/15) from the Israel Science Foundation (ISF) to RK.

## Conflict of interest

The authors declare that the research was conducted in the absence of any commercial or financial relationships that could be construed as a potential conflict of interest.

## Publisher’s note

All claims expressed in this article are solely those of the authors and do not necessarily represent those of their affiliated organizations, or those of the publisher, the editors and the reviewers. Any product that may be evaluated in this article, or claim that may be made by its manufacturer, is not guaranteed or endorsed by the publisher.

## Footnotes

^1^ http://psychtoolbox.org
