# Supplementary material for: Perceptual organization and visual awareness: the case of amodal completion
Source: Front Psychol. 2023 Aug 17;14:1201681. doi: 10.3389/fpsyg.2023.1201681 (PMC10470034; doi:10.3389/fpsyg.2023.1201681)
Supplement: Supplementary file 1 [file Data_Sheet_1.PDF]

Supplemental material

Summary of the results of the repeated measures ANOVA with prime (neutral and occluded) and target (global and local) as within-subject factors, conducted on the reaction time (RT) and accuracy (AC) data.

Experiment 1

Experiment 1a: Invisible prime

RT: Significant main effect of Target, $F(1,26)=4.60$, $p=0.0414$, $\eta_p^2=0.15$; no significant main effect Prime, $F<1$; no significant Target X Prime interaction, $F < 1$.

AC: Significant main effect of Target, $F(1,26)=14.09$, $p=0.0009$, $\eta_p^2=0.35$; no significant main effect Prime, $F(1,26)=4.13$, $p=0.0526$, no significant Target X Prime interaction, $F(1,26)=2.20$, $p=0.1497$.

Experiment 1b: Visible prime.

RT: No significant main effect of Target, $F < 1$; significant main effect of Prime, $F(1,17)=5.97$, $p=0.0357$, $\eta_p^2=0.26$; no significant Target X Prime interaction, $F < 1$.

AC: No significant effects of Target, $F<1$; no significant main effect of Prime, $F(1,17)=2.10$, $p=0.1659$; no significant Target X Prime interaction, $F<1$.

Experiments 2

Experiment 2a: Invisible prime

RT: No significant main effect of Target, $F<1$; no significant main effect Prime, $F<1$; significant Target X Prime interaction, $F(1,26)=6.25$, $p=0.0190$, $\eta_p^2=0.24$.

AC: No significant main effect of Target, $F<1$; significant main effect of Prime, $F(1,26)=5.44$, $p=0.0277$, $\eta_p^2=0.17$; no significant Target X Prime interaction, $F(1,26)=1.00$, $p=0.3265$.

Experiment 2b: Visible prime

RT: No significant main effect of Target, $F(1,17)=2.88$ $p=1080$; no significant main effect Prime, $F(1,17)=3.45$, $p=0.0807$; significant Target X Prime interaction, $F(1,17)=10.86$, $p=0.0043$, $\eta_p^2=0.39$.

AC: no significant main effect of Target, $F<1$; significant main effect of Prime, $F(1,17)=4.48$, $p=0.0494$, $\eta_p^2=0.20$; no significant Target X Prime interaction, $F<1$.

Experiments 3

Experiment 3a: Invisible prime

RT: No significant main effect of Target, $F<1$; no significant main effect Prime, $F(1,26)=2.31$, $p=0.1402$; no significant Target X Prime interaction, $F(1,26)=1.08$, $p= 0.3083$.

AC: No significant main effect of Target, $F<1$; no significant main effect of Prime, $F<1$; no significant Target X Prime interaction, $F<1$.

Experiment 3a: Visible prime

RT: No significant main effect of Target, $F<1$; significant main effect of Prime, $F(1,17)=7.16$, $p=0.0160$, $\eta_p^2=29$; no significant Target X Prime interaction, $F(1,17)=1.00$, $p=0.3305$.

AC: No significant main effect of Target, $F<1$; no significant main effect of Prime, $F(1,17)= 2.27$, $p=0.1500$; no significant Target X Prime interaction, $F < 1$.

Experiment 4

Experiment 4a: Invisible prime

RT: No significant main effect of Target, $F<1$; no significant main effect of Prime, $F<1$; no significant Target X Prime interaction, $F < 1$.

AC: Significant main effect of Target, $F(1,26)=5.58$, $p=0.0259$, $\eta_p^2=18$; no significant main effect of prime, $F<1$; no significant Target X Prime interaction, $F(1,26)=3.52$, $p=0.0719$.

Experiment 4a: Visible prime

RT: No significant main effect of Target, $F<1$; no significant main effect of Prime, $F<1$; significant Target X Prime interaction, $F(1,17)= 8.97$, $p=0.0081$, $\eta_p^2=35$.

AC: No significant main effect of Target, $F(1,17)= 2.17$, $p=0.1594$; no significant main effect of prime, F<1, no significant Target X Prime interaction, $F<1$.
